# Changes in mitochondrial homeostasis and redox status in astronauts following long stays in space

**DOI:** 10.1038/srep39015

**Published:** 2016-12-16

**Authors:** Hiroko P. Indo, Hideyuki J. Majima, Masahiro Terada, Shigeaki Suenaga, Kazuo Tomita, Shin Yamada, Akira Higashibata, Noriaki Ishioka, Takuro Kanekura, Ikuya Nonaka, Clare L. Hawkins, Michael J. Davies, Daret K. St Clair, Chiaki Mukai

**Affiliations:** 1 Department of Oncology and Space Environmental Medicine, Graduate School of Medical and Dental Sciences, Kagoshima University, Kagoshima City, Kagoshima 890-8544, Japan; 2 Divison of Aerospace Medicine, The Jikei University School of Medicine, Minato-ku, Tokyo 105-8461, Japan; 3 Japan Aerospace Exploration Agency, Tsukuba City, Ibaraki 305-8505, Japan; 4 Space Biosciences Division, NASA Ames Research Center, Moffett Field, California 94035, USA; 5 Institute of Space and Astronautical Science, Sagamihara, Kanagawa 252-5210, Japan; 6 Department of Space and Astronautical Science, School of Physical Sciences, SOKENDAI (The Graduate University for Advanced Studies), Sagamihara, Kanagawa 252-5210, Japan; 7 Department of Dermatology, Graduate School of Medical and Dental Sciences, Kagoshima University, Kagoshima City, Kagoshima 890-8544, Japan; 8 National Center Hospital for Mental Nervous and Muscular Disorders, Kodaira, Tokyo 187-8551, Japan; 9 The Heart Research Institute, 7 Eliza Street, Newtown, Sydney, 7 Eliza Street, Newtown, Sydney, NSW 2042, Australia; 10 Sydney Medical School, University of Sydney, Sydney, NSW 2006, Australia; 11 Department of Biomedical Sciences, Panum Institute, University of Copenhagen, Blegdamsvej 3, Copenhagen 2200, Denmark; 12 Department of Toxicology and Cancer Biology, University of Kentucky College of Medicine, Lexington, Kentucky 40536, USA; 13 Tokyo University of Science, Shinjuku, Tokyo 162-0825, Japan

## Abstract

The effects of long-term exposure to extreme space conditions on astronauts were investigated by analyzing hair samples from ten astronauts who had spent six months on the International Space Station (ISS). Two samples were collected before, during and after their stays in the ISS; hereafter, referred to as Preflight, Inflight and Postflight, respectively. The ratios of mitochondrial (mt) to nuclear (n) DNA and mtRNA to nRNA were analyzed via quantitative PCR. The combined data of Preflight, Inflight and Postflight show a significant reduction in the mtDNA/nDNA in Inflight, and significant reductions in the mtRNA/nRNA ratios in both the Inflight and Postflight samples. The mtRNA/mtDNA ratios were relatively constant, except in the Postflight samples. Using the same samples, the expression of redox and signal transduction related genes, *MnSOD, CuZnSOD, Nrf2, Keap1, GPx4* and *Catalase* was also examined. The results of the combined data from Preflight, Inflight and Postflight show a significant decrease in the expression of all of the redox-related genes in the samples collected Postflight, with the exception of *Catalase*, which show no change. This decreased expression may contribute to increased oxidative stress Inflight resulting in the mitochondrial damage that is apparent Postflight.

Cosmic rays were first confirmed by Hess while performing balloon experiments to measure ionizing radiation in the atmosphere^1^. The characteristics of the radiation field in low Earth orbit and in deep space have been previously described^2^,^3^. The details of the particle compositions and energy of these cosmic rays were reported by Simpson in 1983^4^. The dose rate of space radiation has been estimated to be 0.1–1.0 mGy/day, over one hundred times more than that experienced on Earth^5^. Therefore, the risks posed by space radiation exposure to astronaut health should be of concern^6^,^7^,^8^,^9^.

In addition to space radiation, microgravity is also expected to affect health and normal cellular homeostasis. Many lines of evidence suggest that conditions experienced during microgravity result in increased oxidative stress^10^,^11^. Indo *et al*. examined changes in 15 apoptosis-related genes, 1 autophagy-related gene and 1 necrosis-related gene using quantitative real-time reverse transcription polymerase chain reaction (qRT-PCR) analyses following 24 hours of simulated microgravity in a human neuroblastoma cell line, NB-1, which expresses the wild-type *p53*^12^. The study demonstrated changes in 3 genes: an upregulation of the proapoptotic voltage-dependent anion channel 2 (*VDAC2*) gene and the downregulation of the proapoptotic caspase-9 (*CASP9*) and antiapoptotic manganese superoxide dismutase (*MnSOD*) genes. These results suggest that microgravity may decrease apoptosis but may potentially increase oxidative stress. Wang indicated that simulated microgravity promotes PC12 cell senescence as a result of increases in oxidant stress^13^.

Mitochondria are the largest source of energy production in the cell, which occurs through oxidative phosphorylation (OXPHOS), and many findings suggest that mitochondria also play an important role in apoptosis; this is known as mitochondria-mediated cell death^14^,^15^,^16^,^17^,^18^. Mitochondria control not only apoptosis but also necrosis and autophagy^19^. It has been reported that electron leakage (2–3%) from the mitochondrial electron transport chain and the reaction of these electrons with oxygen results in the generation of superoxides, which initiate oxidative stress^20^,^21^. In addition, the mitochondrial generation of reactive oxygen species (ROS) relates to the aging process^22^. Thus, exposure to space environments, which are characterized by space radiation and microgravity, may increase mitochondria-related oxidative stress and subsequent cell death^19^.

Barrientos *et al*. demonstrated that the amount of mitochondrial RNA (mtRNA) remains relatively constant and is not dependent on age, which is due to age-dependent increases in the levels of mitochondrial DNA (mtDNA) and age-dependent decreases in mitochondrial DNA transcription rates^22^. Therefore, biogenesis and the maintenance of mitochondrial DNA and RNA are likely important in controlling homeostasis. Many factors influence cellular homeostasis and health under different conditions, especially during exposure to different stresses, such as those encountered in space. Reduction–oxidation reaction (Redox) status should be also considered.

In 2009, the Japan Aerospace Exploration Agency (JAXA) initiated a clinical investigation to study the effects of long-term spaceflight on gene expression and mineral metabolism by analyzing hair samples from International Space station (ISS) crew members that have been in space (experiment nicknamed “HAIR”). Ten astronauts from the ISS crew participated in the HAIR experiment. This study was initiated according to a treaty between JAXA and Kagoshima University. This study complies with the terms of the treaty and was performed using methods that were carried out in accordance with the approved guidelines.

## Results

**Figure 1:**
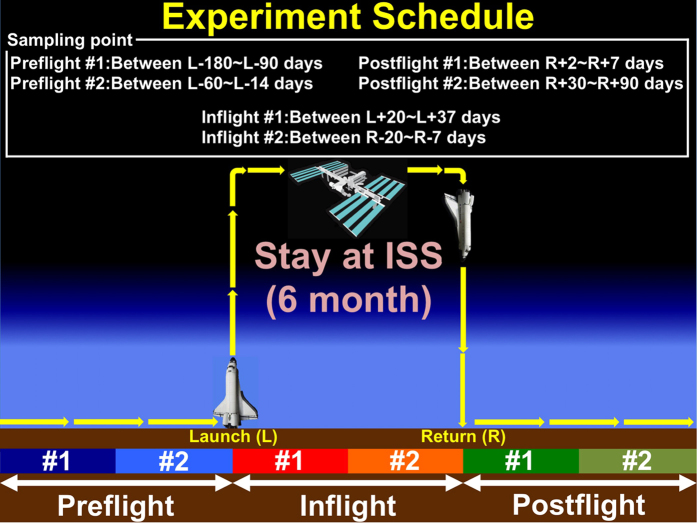
Astronaut’s hair sampling schedule. At each sampling stage, two astronauts extracted their hair strands (sample number indicated in the text). L: Launch, R: Return. Two x 3 periods of astronaut hair strands were used in this study. The first time a sample was taken was designated #1, and the second time a sample was taken was designated #2 for each Preflight, Inflight and Postflight period. Preflight #1: L-180~L-90 days (6~3 months before launch), Preflight #2: L-60~L-14 (from 2 months to 2 weeks before launch), Inflight #1: L + 20~L + 37 (from 20 to 37 days after launch), Inflight #2: R-20~R-7 (from 20 to 7 days before return), Postflight #1 R + 2~R + 7 (from 2 to 7 days after return), and Postflight #2 R + 30~R + 90 (from 1 to 3 months after return). L: Launch, R: Return.

A scheme detailing hair sample preparation is listed in Fig. 1. As described earlier, mtDNA encodes 13 genes, of which the resulting proteins are a part of the ETC that produces ATP via OXPHOS. Therefore, information relating to mtDNA and mtRNA production is important to evaluate cellular homeostasis. In this study, DNA and RNA were extracted from cells in hair bulbs, which were taken from ten astronauts as described earlier. To obtain adequate amounts of mtDNA and mtRNA, we adjusted the amounts based on nDNA and nRNA. We determined Preflight, Inflight and Postflight mtDNA/nDNA, mtRNA/nRNA and mtRNA/mtDNA ratios as shown in the Materials and Methods section of the Supplementary Information. The mtDNA/nDNA, mtRNA/nRNA and mtRNA/mtDNA ratios from samples taken Preflight #1, #2, Inflight #1, #2 and Postflight #1, #2 for each astronaut are shown in Supplementary Fig. S1a–c. The data from each astronaut show relatively different patterns. Increased or decreased mtDNA/nDNA, mtRNA/nRNA, and mtRNA/mtDNA ratios for ten astronauts obtained from data in Supplementary Fig. S1 are summarized in Supplementary Fig. S2. As shown in Supplementary Fig. S2, there are some clear trends in the changes to the mtDNA/nDNA and mtRNA/nRNA ratios, while there is a less clear trend for the mtRNA/mtDNA ratio, except in the comparison of Inflight vs Postflight results.

**Figure 2:**
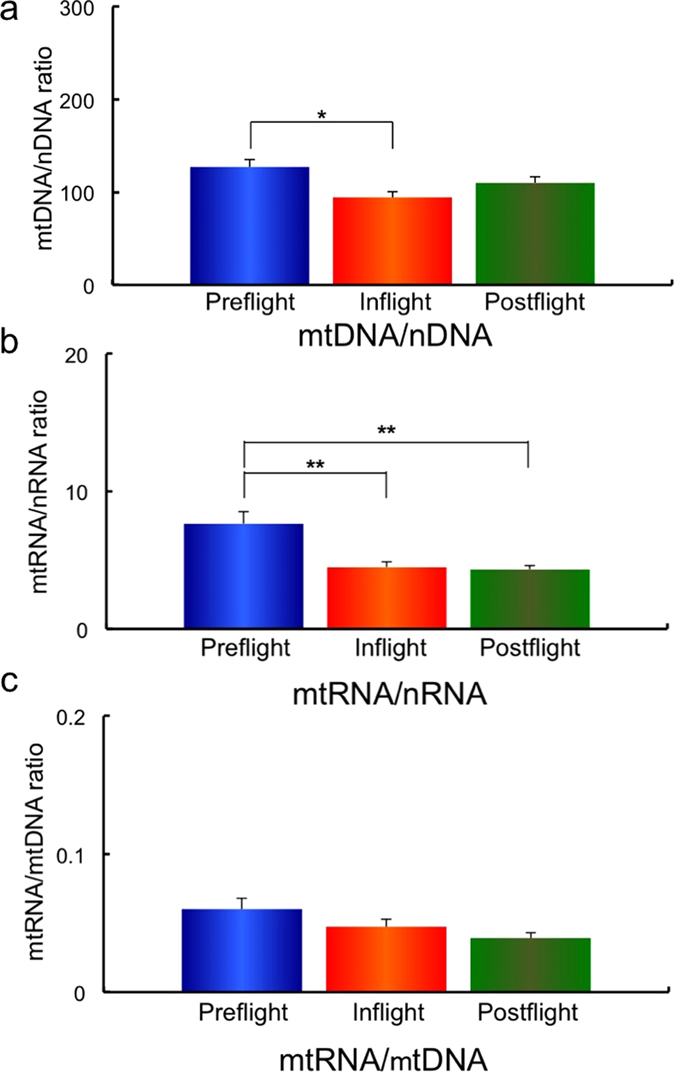
Combined data for the mtDNA/nDNA (**a**), mtRNA/nRNA (**b**) and mtRNA/mtDNA (**c**) ratios from ten astronauts in comparison with Preflight, Inflight and Postflight. Mean data of the mtDNA/nDNA (**a**), mtRNA/nRNA (**b**) and mtRNA/mtDNA (**c**) ratios for all 10 astronauts are shown. The mtRNA/mtDNA ratios shown in (**c**) were calculated from data of mtRNA/nRNA and mtRNA/mtDNA according to the equation as shown in Materials and method. *p ≤ 0.05 by ANOVA with Scheffe’s F test, **p ≤ 0.01 by ANOVA with Scheffe’s F test. Bar: SE.

The combined data of mtDNA/nDNA, mtRNA/nRNA, and mtRNA/mtDNA ratios for Preflight #1, #2, Inflight #1, #2 and Postflight #1, #2 for all ten astronauts are shown in Supplementary Fig. S9a–c. Comparisons of the mtDNA/nDNA, mtRNA/nRNA, and mtRNA/mtDNA ratios in Preflight, Inflight and Postflight are shown in Fig. 2. A significant reduction of mtDNA/nDNA ratio is observed on comparing the samples taken Preflight with those taken Inflight (Fig. 2a). Similarly, a significant reduction of mtRNA/nRNA in Inflight and Postflight samples is seen compared to the levels in samples taken Preflight (Fig. 2b). There is no significant change in the mtRNA/mtDNA ratio between Preflight, Inflight and Postflight as shown in Fig. 2c.

**Figure 3:**
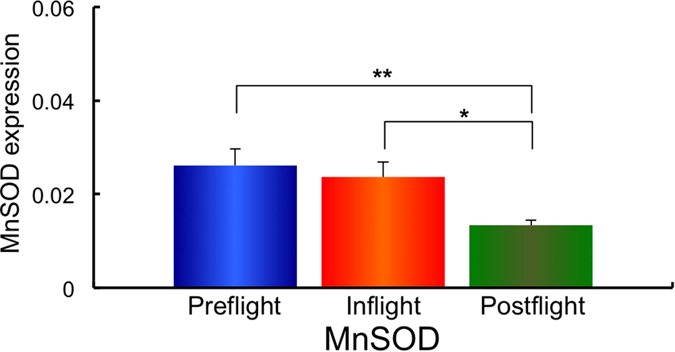
Combined data for MnSOD expression changes in comparison with Preflight, Inflight and Postflight. Mean data of MnSOD expression change for all 10 astronauts are shown. *p ≤ 0.05 by ANOVA with Scheffe’s F test, **p ≤ 0.01 by ANOVA with Scheffe’s F test. Bar: SE.

**Figure 4:**
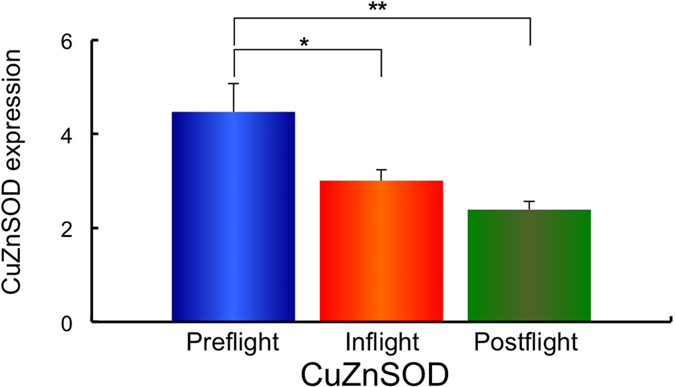
Combined data for CuZnSOD expression changes in comparison with Preflight, Inflight and Postflight. Mean data of CuZnSOD expression change for all 10 astronauts are shown. *p ≤ 0.05 by ANOVA with Scheffe’s F test, **p ≤ 0.01 by ANOVA with Scheffe’s F test. Bar: SE.

**Figure 5:**
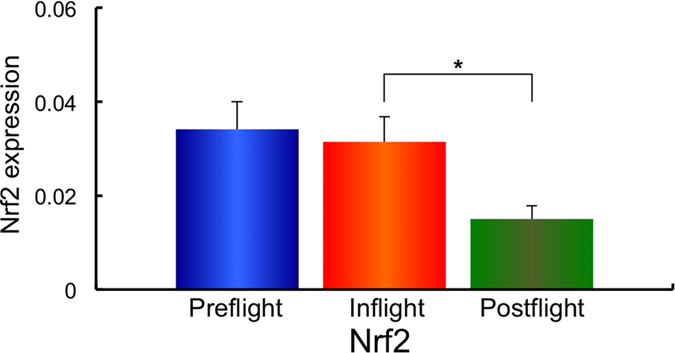
Combined data for Nrf2 expression changes in comparison with Preflight, Inflight and Postflight. Mean data of Nrf2 expression change for all 10 astronauts are shown. *p ≤ 0.05 by ANOVA with Scheffe’s F test, **p ≤ 0.01 by ANOVA with Scheffe’s F test. Bar: SE.

**Figure 6:**
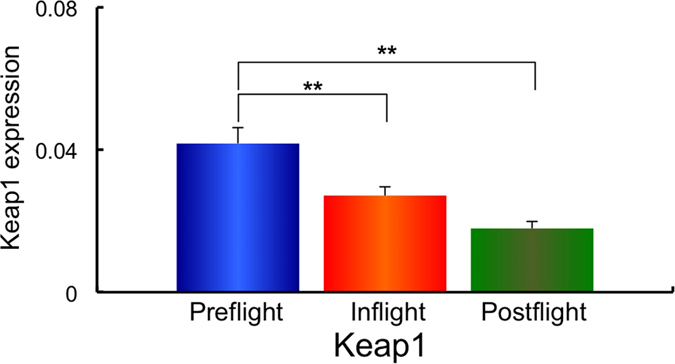
Combined data for Keap1 expression changes in comparison with Preflight, Inflight and Postflight. Mean data of Keap1 expression change for all 10 astronauts are shown. *p ≤ 0.05 by ANOVA with Scheffe’s F test, **p ≤ 0.01 by ANOVA with Scheffe’s F test. Bar: SE.

**Figure 7:**
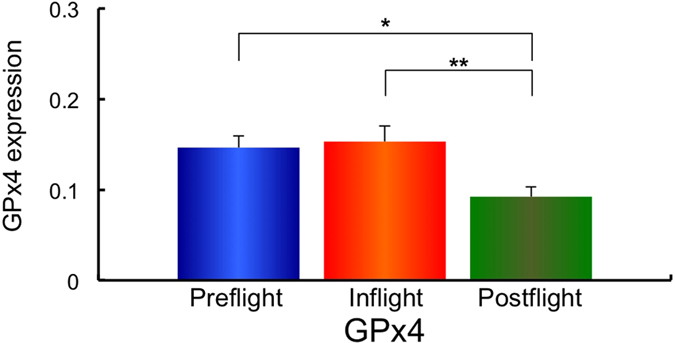
Combined data for GPx4 expression changes in comparison with Preflight, Inflight and Postflight. Mean data of GPx4 expression change for all 10 astronauts are shown. *p ≤ 0.05 by ANOVA with Scheffe’s F test, **p ≤ 0.01 by ANOVA with Scheffe’s F test. Bar: SE.

**Figure 8:**
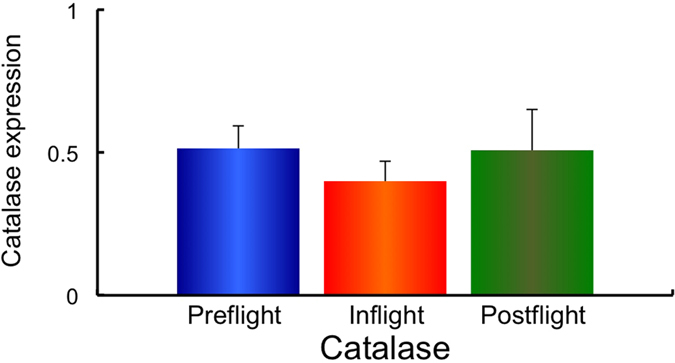
Combined data for Catalase expression changes in comparison with Preflight, Inflight and Postflight. Mean data of Catalase expression change for all 10 astronauts are shown. *p ≤ 0.05 by ANOVA with Scheffe’s F test, **p ≤ 0.01 by ANOVA with Scheffe’s F test. Bar: SE.

Next, using the same samples, the gene expression of the redox-sensitive enzymes mitochondrial manganese superoxide dismutase (*MnSOD*), and the cytosol enzymes, copper zinc superoxide dismutase (*CuZnSOD*), Nuclear factor (erythroid-derived 2)-like 2 (*Nrf2*), Kelch-like ECH-associated protein 1 (*Keap1*), Glutathione peroxidase 4 (*GPx4*) and Catalase was examined. The expression of *MnSOD, CuZnSOD, Nrf2, Keap1, GPx4* and Catalase in Preflight #1, #2, Inflight #1, #2 and Postflight #1, #2 for each astronaut are shown in Supplementary Figs S3–S8. The combined data of MnSOD, CuZnSOD, Nrf2, Keap1, GPx4 and Catalase expressions from all ten astronauts in Preflight #1, #2, Inflight #1, #2 and Postflight #1, #2 data are shown in Supplementary Figs S10–S15. Comparisons of *MnSOD, CuZnSOD, Nrf2, Keap1, GPx4* and *Catalase* expression in Preflight, Inflight and Postflight samples from the combined data of ten astronauts are shown in Figs 3–8. The results show that the expression of the redox-related genes are significantly reduced in Postflight samples compared to samples collected Preflight and Inflight, with the exception of *Catalase*, which was unchanged across each condition.

## Discussion

### Hair follicle as a useful tool in analyzing biogenesis

We previously discussed the usefulness of hair follicles in the study of biogenesis^23^. Our group reported that in some astronauts from the same group examined in this study, genes related to hair growth, such as the fibroblast growth factor (FGF) 18, angiopoietin-like 7 (ANGPTL7) and cartilage oligomeric matrix protein (COMP), were upregulated Inflight. We suggested that spaceflight inhibits cell proliferation in hair follicles^24^. Hair follicles reconstitute themselves during the hair growth cycle, suggesting the presence of intrinsic stem cells^25^. Hair samples can be easily taken and be used as a tool for monitoring health^26^. It has been previously shown that cells in the hair follicle are much more sensitive to radiation compared with epidermal cells, and that this sensitivity is p53 dependent^27^. After receiving 6 Gy of X-ray irradiation, the normal structure of the hair bulb is totally abrogated, although the epithelial tissues associated with papillary cells remain, suggesting that cells of the hair bulb are radiation sensitive^28^. Therefore, the hair bulb is an appropriate tool for studying the effects of space radiation, which may influence health.

### Observations of the mitochondrial RNA and DNA ratios provide indicators of homeostatic changes in an individual

Many lines of study have used the housekeeping gene, nuclear coded glyceraldehyde-3-phosphate dehydrogenase (*GAPDH*), as a nuclear control gene in microgravity experiments^29^,^30^, and *GAPDH* expression does not show differences between ground control and microgravity treatments^31^,^32^. To improve the precision of the data, we normalized our data to the levels of nDNA and nRNA to determine changes in mtDNA and mtRNA content in the Preflight, Inflight and Postflight samples (Fig. 2).

The importance of mitochondrial biogenesis in maintaining health, the development of diseases and aging has been described^33^,^34^,^35^. The initiation of transcription in mammalian mitochondria depends on three proteins: mitochondrial RNA polymerase (*POLRMT*), mitochondrial transcription factor A (*TFAM*) and mitochondrial transcription factor B2 (*TFB2 M*), which are nuclear encoded genes^36^. Therefore, cellular metabolism influences mitochondrial transcription.

Mitochondrial DNA is known to encode seven subunits of complex I (NADH dehydrogenase), one unit of coenzyme Q-cytochrome *c* reductase/cytochrome *b* (complex III), three subunits of cytochrome *c* oxidase (complex IV), and two subunits of ATP synthase^37^. The transcription and translation of these genes proceeds in the mitochondrial matrix independently of the processes of nuclear DNA^37^,^38^. Even if the temperature of the environment is changed, the production of mtDNA and mtRNA is not changed^39^. It is known that increases in mtDNA production compensate for the reduced production of mtRNA that occurs with aging^40^. We used the NADH-ubiquinone oxidoreductase chain 1 (*ND1*) gene for analyzing mtDNA and mtRNA, which was performed via qRT-PCR. The ND1 protein is part of a large enzyme complex known as complex I. Complex I is one of several enzyme complexes necessary for OXPHOS.

Exercise results in damage in mitochondria^41^. It is known that mitochondrial DNA and RNA production differs between developing and adult rats^42^ and aging causes malfunctions in mitochondria^20^. Previously, we have published that mitochondria in cells with impaired electron transport chain and mitochondrial DNA damage cause increase of mitochondrial ROS generation^21^. Change of levels of mtDNA production may increase the generation of mitochondrial ROS. Corticotropin, which induces the secretion of other hormones, including corticosterone, and vitamin 12 are involved in regulating mtDNA and mtRNA expression^43^,^44^. Gadaleta *et al*. reported that treatment with triiodothyronine, a thyroid hormone, resulted in increases in mtDNA transcription^45^. Thus, changes to health status likely change the mtDNA and mtRNA content.

### Mitochondrial DNA and RNA homeostasis change in space

In space, living conditions are different than on Earth. Alterations in gravity can influence homeostasis and health status, and space radiation is also a major factor and potential inducer of stress during long stays in space. In this study, hair bulb samples were examined to determine nuclear and mitochondrial DNA and RNA ratios because mitochondrial DNA and RNA contribute to cellular energy production, homeostasis and may also reflect the induction of oxidative stress. We have demonstrated previously that ROS generation from mitochondria controls lipid peroxidation and apoptosis, which can be detrimental to cellular function and act as a potential contributor to disease^20^.

Doses of radiation in space are over one hundred times greater than the doses received on the surface of the Earth. In addition to the effects of space radiation, the effects of isolation and microgravity can also affect the activity and emotions of astronauts. Working and living in specific locations, especially under the different conditions experienced in space, can cause stress in humans, although astronauts have been highly selected from the general population. This study examines results from ten astronauts, who stayed in the ISS for a long period. Understanding the biological situations involved with prolonged stays in space will provide valuable information for dealing with the differences between lives on Earth and in space. The observation of this study shows no change in the mtRNA.mtDNA ratio (Fig. 2c). However, mtDNA/nDNA ratio was decreased on comparing Preflight and Inflight samples as shown in Fig. 2a. Similarly, mtRNA.nRNA ratio decreased in Inflight and Postflight compared to Preflight. This may imply that the cells in the hair follicle received damage due to space radiation, with a reduced number of cells recovered in Postflight, but the damage, in terms of mtDNA transcription, remained comparable to Postflight as shown in Fig. 2b.

### Effects of the space environment on health status

Several studies suggest that microgravity affects cellular functions such as proliferation^46^, signal transduction^47^ and gene expression^48^. Stein *et al*. examined energy intake and protein synthesis in two astronauts (American) and four cosmonauts (Russian) during long-duration (over 3 month) flights on the Russian space station MIR^49^. They observed that energy intake and protein synthesis decreased in a correlated manner. After the flight, energy intake and protein synthesis levels returned to the preflight levels. Stein and Leskiw further examined oxidative damage and urinary excretion of F2 isoprostane and 8-hydroxydeoxyguanosine (8-OHdG) during and after spaceflight for the same two astronauts and four cosmonauts^50^. Samples were obtained from MIR participants inflight between 88 and 186 days in orbit. F2 isoprostane, 8-iso-Prostaglandin F_2_α (8-iso-PGF_2_α) and 8-OHdG were used as markers for oxidative damage to membrane lipids and DNA, respectively. The results show that on the shuttle, 8-OHdG excretion was unchanged inflight and postflight, although 8-iso-PGF_2_α excretion was decreased inflight (p ≤ 0.05). The changes in 8-iso-PGF_2_α production were attributed to decreases in the production of oxygen radicals from the electron transport chain due to the reduced energy intake inflight. They suggested that postflight increases in the excretion of the products of oxidative damage were attributed to a combination of an increase in metabolic activity and the loss of some host antioxidant defenses inflight. They concluded that oxidative damage was decreased inflight and increased postflight^50^. Increases in oxidative stress associated with spaceflight may persist after a return to normal gravity, and this effect may be more pronounced after an extended period of time spent in space^10^. Hollander *et al*. examined liver antioxidant enzyme activities, mRNA abundance, and glutathione (GSH) status in male Sprague–Dawley rats placed in an enclosed module aboard the Space Shuttle STS-63 for 8 days (F, n = 6)^51^. Spaceflight significantly reduced the total GSH content and the activities of copper zinc SOD (CuZnSOD), catalase, GSH reductase, and GSH sulfur-transferase in the liver. These results indicate that spaceflight can induce a downregulation of the antioxidant defense system, which is also observed in the current study, where a significant decrease in the expression of various redox-related enzymes was seen Inflight and Postflight. In the former case, this downregulation promoted oxidative stress in the liver^51^. Degan *et al*. reported that exposure to simulated microgravity reduces intracellular ATP concentrations in freshly drawn lymphocytes and lymphoblastoid cells by approximately 40−50% of normal levels^52^. They also found that poly(ADP-ribose) polymerase (PARP) activity increases, indicating that cells exposed to reduced gravity are stressed. Maillet *et al*. reviewed the effects of microgravity in terms of an accelerated model of nutritional disturbances, and concluded that adopting an integrated approach will be essential for optimizing the health of astronauts^53^. In the Space Shuttle STS-76 experiment, Schatten *et al*. found apoptosis and mitochondrial morphological changes in response to space exposure for 4 and 48 hours using acute T-cell leukemia Jurkat cells^54^,^55^. It has been noted that because experiments conducted in space are performed under conditions involving microgravity and space radiation, these findings might be caused by both factors.

### Effects of other factors on mtDNA and mtRNA production

The conditions experienced and the hardware required to live in space will be of great concern for future space travel. Temperature, carbon dioxide levels and humidity are well controlled in space shuttles^56^,^57^. A project examining immune parameters, i.e., peripheral leukocyte distribution, T-cell function, virus-specific immunity, and mitogen-stimulated cytokine production profiles, in twenty-three astronauts revealed that the immune alterations persist during long-duration (6 month) spaceflights^58^. Immune alterations are likely to be critical factors in the changes in homeostasis occurring in astronauts. In addition, several other factors are likely to influence astronauts, including mental or psychological, philosophical, and physical factors during the preflight, inflight and postflight periods. Emotions involved in the first trip to space, changes in sleeping time and fatigue can influence the health of astronauts in the preflight period. The completely different conditions of microgravity, altered diets, different circadian rhythms and stress management might also influence astronaut health during spaceflight. In addition, space radiation could influence astronaut health. After the flight, emotional, psychological and physical effects, as well as changes to eating and circadian cycles and gravity could again influence an astronaut’s health. Factors that influence astronauts and the possible aging effects of space travel have been described by the former astronauts Vernicos and Schneider^59^. Nutelings *et al*. suggested that three months of exposure to space conditions induced skin atrophy and deregulated hair growth in mice that were maintained in the Mice Drawer System (MDS)^60^ for 91 days aboard the ISS^61^. Another recent study conducted in space during the STS-135 mission, where female C57BL/6 mice survived for 13 days, resulted in increased oxidative stress in the skin, which was assessed by examining 4-hydroxynonenal (4-HNE)^62^.

### Changes of oxidative stress related gene expressions in Preflight, Inflight and Postflight

The expression of six genes, *MnSOD, CuZnSOD, Nrf2, Keap1, GPx4* and *Catalase* were examined in Preflight, Inflight and Postflight samples in this study. MnSOD is an enzyme that catalyses the dismutation of superoxide, which is formed by oxygen and electrons that are released from electron transport systems (ETC), to hydrogen peroxide. Mitochondria are a major source of reactive oxygen species (ROS), particularly superoxide, which can be transformed to other reactive species that promote oxidative stress and related diseases^20^. CuZnSOD is localized in cytosol, and is also responsible for the dismutation of superoxide^63^. Alterations in the level of ROS within cells causes the perturbation of signaling processes, with the Nrf2 – Keap1 system recognized as a major signaling pathway to protect cells from oxidative stress^64^. Human Keap1 has 27 cysteines, with oxidation of cysteine resulting in a conformational change that causes the release of Keap1 from bound Nrf2, enabling nuclear translocation and activation of Nrf2. Nrf2 plays an important transductive signaling role that causes upregulation of the cellular antioxidant defenses^64^. GPx4 is a phospholipid hydroperoxide that protects cells against membrane lipid peroxidation, and their isoforms are produced by alternative splicing and transcription initiation. There is evidence to support the localization of GPx4 to the mitochondria^65^. Catalase is a cytosolic enzyme responsible for the removal of hydrogen peroxide (H_2_O_2_). Thus, together, these proteins play a key role in maintaining the cellular redox balance. Our results show that *CuZnSOD* and *Keap1* genes, together with the mtDNA/nDNA and mtRNA/nRNA are downregulated in Inflight, supporting the suggestion that cells in the hair follicle are damaged by exposure to space radiation in Inflight. The mtRNA/nRNA ratio was also decreased Postflight, as was the expression of *CuZnSOD* and *Keap1*. Interestingly, the gene expression of *MnSOD, Nrf2* and *GPx4* was further downregulated Postflight compared with Inflight, suggesting sustained damage to the cells of hair follicle, which is again supported by the reduced mtRNA/nRNA ratios from Postflight compared to Preflight samples.

The conclusions from this study are that a prolonged exposure to space conditions may induce reduced mtDNA and mtRNA production and that this phenomenon continues even after spaceflight, based on an examination of samples from ten astronauts who stayed in the ISS for a period of six months. Our previous study^24^ conducted using DNA array and qRT-PCR analyses using the same samples of astronauts concluded that cell proliferation in cell follicles was inhibited Inflight. The results presented in this study showing reduced mtDNA and mtRNA expression is supported by data showing decreased expression of several key redox-regulated enzymes. These results may be due to the high radiation sensitivity of hair follicle cells. The effects of longer stays in space stay, such as those involved in going to Mars, are therefore of great concern. These types of trips may have a more significant influence on cells of important organs, such as neurons.

## Methods

### Ethics Statement

This study was carried out in accordance with the guidelines approved by the Committee on Human Care and Use by the NASA and JAXA Ethical Review Board and the Human Research Multilateral Review Board (HRMRB). All participants provided written informed consent.

### Hair sample preparation

Ten astronauts at the International Space Station (ISS) participated in the study. They were at the ISS on a 6-month-long mission. During each mission, five strands of hair were sampled six times from each astronaut. The 6 different sampling times were as follows (Fig. 1): first Preflight (Launch (L)-180~90 days: 6~3 months before launch), second Preflight (L-60~14: from 2 months to 2 weeks before launch), first Inflight (L + 20~37: from 20 to 37 days after launch), second Inflight (Return (R)-20~7: from 20 117 to 7 days before return), first Postflight (R + 2~7: from 2 to 7 days after return), and second Postflight (R + 30~90: from 1 to 3 months after return). The sampling days differed for each astronaut because of differing schedules. In each mission, two astronauts were paired, and individual hair samples were collected. For each individual sample, five strands of hair were grasped as close as possible to the scalp and pulled out using tweezers in the direction of hair growth without damaging the hair roots. The Preflight and Postflight samples were stored at −80 °C until analysis. The Inflight samples were stored in the Minus Eighty Degree Laboratory Freezer on the ISS (MELFI) as soon as possible after collection until being returned for analysis.

### RNA extraction

Hair roots (approximately 2–3 mm) were used as the source for extracted mRNA. The roots were cut into approximately 15 fragments (0.1–0.2 mm each) by using a microsurgical knife under a stereoscopic microscope. The collected fragments were immersed in 800 μl of ISOGEN reagent (Nippon Gene; Toyama, Japan) in tubes and stirred (15 sec × 2 times) using a Bioruptor UCD-250 sonication device (Cosmo Bio; Tokyo, Japan). Next, the RNA was purified from hair lysates using an ISOGEN kit according to the manufacturer’s instructions. Briefly, the tubes were maintained at room temperature for 5 min, followed by the addition of 200 μl of chloroform. The subsequent process of RNA purification was performed according to the manufacturer’s instructions. After isolation, RNA pellets were washed with 70% ethanol, air dried, and resuspended in 10 μl of RNA-free water (Gibco-BRL; Gaithersburg, MD). Total RNA was quantified at 260 nm using a NanoDrop ND-1000 spectrophotometer (NanoDrop Technologies Inc.; Wilmington, DE). RNA quality was determined using an Agilent Bioanalyzer 2100 (Agilent Technologies; Palo Alto, CA). The 28S:18S rRNA ratio and the RNA integrity number (RIN) were calculated using the 2100 Expert and RIN Beta Version software (Agilent Technologies), respectively.

### RNA amplification

As the RNA sample extracted from the hair samples was small, a double RNA amplification step was incorporated prior to microarray hybridization. Total RNA was amplified using an Ambion MessageAmp aRNA Kit (Thermo Fisher Scientific; Waltham MA, USA). Briefly, first- and second-strand cDNA were synthesized. Unlabeled aRNA was generated by *in vitro* transcription with non-biotinylated NTPs. For probe preparation, RNA was reverse-transcribed with second-round primers. The second-strand cDNA was synthesized with T7 oligo(dT) primers and purified. Biotin-labeled cRNA was generated via *in vitro* transcription and purified using an RNeasy Kit (Qiagen; Venlo, Netherlands).

### DNA extraction from hair RNA samples

DNA samples from the astronauts’ hair roots were extracted using ISOGEN (Nippon Gene; Toyama, Japan) from the rest of the extracted RNA samples according to the manufacturer’s instructions. Briefly, 0.3 ml of 100% ethanol were added to each sample, followed by a 3-minute incubation at room temperature. Following centrifugation using a refrigerated microcentrifuge (Kubota model 3500; KUBOTA Tokyo, Japan) at 2000 *g* and 4 °C for 5 minutes, the precipitate in the tubes was washed with 1 ml of 0.1 M sodium citrate in 10% ethanol for 30 minutes. After further centrifugation at 2000 *g* and 4 °C for 5 minutes, precipitates were washed again with 1 ml of 0.1 M sodium citrate in 10% ethanol for another 30 minutes. The isolated DNA pellets were washed with 1 ml of ethanol and then centrifuged at 2000 *g* and 4 °C for 5 minutes. The rinsed DNA samples were air dried and re-suspended in 50 μl of Tris-ethylenediaminetetraacetic acid (EDTA) buffer solution (TE solution). To quantify the amount of DNA, the absorbance rates at 260 nm were measured using a U-1900 spectrophotometer (Hitachi High-Tech Science; Tokyo, Japan). The 260 nm/280 nm absorbance ratio of the DNA samples was also measured to determine DNA quality.

### Measurement of mtDNA/nDNA, mtRNA/nRNA and mtRNA/mtDNA ratios

The ratio of mitochondrial (mt) and nuclear (n) DNA and the ratio of mtRNA and nRNA from the astronauts’ hair roots were analyzed via SYBR Green-based quantitative PCR (qPCR). The DNA and RNA samples described above were used for qPCR. Ten nanograms of DNA were used to detect mt or nDNA, and 2 ng of RNA was used to detect mt or nRNA. The qPCR reactions were performed with the ABI Prism 7000 sequence detection system (Applied Biosystems; Foster City, CA, USA) using a QuantiTect SYBR Green PCR Kit (QIAGEN; Valencia, CA, USA) as recommended by the manufacturer, except for the following modifications. For *ND1* and *GAPDH* amplification, the denature temperature was 95 °C, the denature time was 15 seconds, the annealing temperature was 55 °C, the annealing time was 30 seconds, the elongation temperature was 72 °C and the elongation time was 40 seconds.

NADH dehydrogenase subunit 1 (*ND1*) primers were used to detect mtDNA or mtRNA, and glyceraldehyde-3-phosphate dehydrogenase (*GAPDH*) primers were used to detect nDNA or nRNA. These primer sequences are as follows.

*ND1* F: 5′-ACCCCCGATTCCGCTACGACCAAC-3′,

*ND1* R: 5′-GGTTTGAGGGGGAATGCTGGAGAT-3′,

*GAPDH* F: 5′-GGGCAAGGTCATCCCTGAGCTGAA-3′,

*GAPDH* R: 5′-TCTAGACGGCAGGTCAGGTCCACC-3′.

### Calculation of the mtDNA/nDNA, mtRNA/nRNA, and mtRNA/mtDNA ratios

The mtDNA/nDNA, mtRNA/nRNA, and mtRNA/mtDNA ratios were calculated using the equations below. The threshold cycle (Ct) is defined as “the fractional cycle number at which the fluorescence generated by SYBR Green passes above a fixed threshold.”

Ratio of mtDNA/nDNA and ratio of mtRNA/nRNA = 2^(GAPDH Ct- ND1 Ct).

Ratio of mtRNA/mtDNA = (mtRNA/nRNA)/(mtDNA/nDNA).

### Measurements of MnSOD, CuZnSOD, Nrf2, Keap1, GPx4 and Catalase gene expressions

The qPCR reactions were performed with the ABI Prism 7000 sequence detection system (Applied Biosystems; Foster City, CA, USA) using a QuantiTect SYBR Green PCR Kit (QIAGEN; Valencia, CA, USA). *GAPDH* was used for the control. PCR conditions were as follows: For *MnSOD, Nrf2, Keap1, GPx4* amplification, the denature temperature was 95 °C, the denature time was 15 seconds, the annealing and elongation temperature was 60 °C, the annealing and elongation time was 1 minute. For *CuZnSDO* amplification, the denature temperature was 95 °C, the denature time was 30 seconds, the annealing temperature was 55 °C, the annealing time was 30 seconds, the elongation temperature was 72 °C and the elongation time was 30 seconds. For *Catalase* amplification, the denature temperature was 95 °C, the denature time was 10 seconds, the annealing temperature was 55 °C, the annealing time was 30 seconds, the elongation temperature was 72 °C and the elongation time was 40 seconds. GAPDH was used as control amplification. These primer sequences are as follows.

*MnSOD* F: 5′-TTCTGGACAAACCTCAGCCCTAACGGT-3′

*MnSOD* R: 5′-AACAGATGCAGCCGTCAGCTTCTCCTTAAA-3′

*CuZnSOD* F: 5′-CATTGCATCATTGGCCGCACACTG-3′

*CuZnSOD* R: 5′-ACCACAAGCCAAACGACTTCCAGC-3′

*Nrf2* F: 5′-GTGGCTGCTCAGAATTGCAGAAAAAGAAAA-3′

*Nrf2* R: 5′-TGTTTTTTCAGTAGGTGAAGGCTTTTGTCA-3′

*Keap1* F: 5′-CCATGAAGCACCGGCGAAGTGCC-3′

*Keap1* R: 5′-GTCTGTATCTGGGTCGTAACACTCCAC-3′

*GPx4*F: 5′-GAGCCAGGGAGTAACGAAGAGATCAAA-3′

*GPx4*R: 5′-TCACGCAGATCTTGCTGAACATATCGAATT-3′

*Catalase* F: 5′-TGACTACGGGAGCCACATCCAGGC-3′

*Catalase* R: 5′-TCACAGATTTGCCTTCTCCCTTGC-3′

*GAPDH* F: 5′-GGGCAAGGTCATCCCTGAGCTGAA-3′,

*GAPDH* R: 5′-TCTAGACGGCAGGTCAGGTCCACC-3′.

### Statistical analysis

Statistical analyses were performed using Scheffe’s F test. All *p* values less than 0.05 were considered to be statistically significant.

## Additional Information

**How to cite this article**: Indo, H. P. *et al*. Changes in mitochondrial homeostasis and redox status in astronauts following long stays in space. *Sci. Rep.* **6**, 39015; doi: 10.1038/srep39015 (2016).

**Publisher's note:** Springer Nature remains neutral with regard to jurisdictional claims in published maps and institutional affiliations.

## Supplementary Material

Supplementary Figures
