# Author Correction: Changes in mitochondrial homeostasis and redox status in astronauts following long stays in space

**DOI:** 10.1038/s41598-020-63753-7

**Published:** 2020-04-20

**Authors:** Hiroko P. Indo, Hideyuki J. Majima, Masahiro Terada, Shigeaki Suenaga, Kazuo Tomita, Akira Higashibata, Noriaki Ishioka, Takuro Kanekura, Clare L. Hawkins, Michael J. Davies, Daret K. St Clair, Chiaki Mukai

**Affiliations:** 1 Department of Oncology and Space Environmental Medicine, Graduate School of Medical and Dental Sciences, Kagoshima University, Kagoshima City, 890-8544 Kagoshima Japan; 2 Divison of Aerospace Medicine, The Jikei University School of Medicine, Minato-ku, 105-8461 Tokyo Japan; 3 Japan Aerospace Exploration Agency, Tsukuba City, 305-8505 Ibaraki Japan; 4 Space Biosciences Division, NASA Ames Research Center, Moffett Field, California, 94035 USA; 5 Institute of Space and Astronautical Science, Sagamihara, 252-5210 Kanagawa Japan; 6 Department of Space and Astronautical Science, School of Physical Sciences, SOKENDAI (The Graduate University for Advanced Studies), Sagamihara, 252-5210 Kanagawa Japan; 7 Department of Dermatology, Graduate School of Medical and Dental Sciences, Kagoshima University, Kagoshima City, 890-8544 Kagoshima Japan; 8 The Heart Research Institute, 7 Eliza Street, Newtown, Sydney, 7 Eliza Street, Newtown, Australia; 9 Sydney Medical School, University of Sydney, Sydney, 2006 NSW Australia; 10 Department of Biomedical Sciences, Panum Institute, University of Copenhagen, Blegdamsvej 3, Copenhagen, 2200 Denmark; 11 Department of Toxicology and Cancer Biology, University of Kentucky College of Medicine, Lexington, 40536 Kentucky USA; 12 Tokyo University of Science, Shinjuku, 162-0825 Tokyo Japan

Correction to: *Scientific Reports* 10.1038/srep39015, published online 16 December 2016

An institutional investigation at the JAXA Human Spaceflight Technology Directorate concluded that Ikuya Nonaka and Shin Yamada did not make contributions to writing the paper, analysing the data, or checking and confirming the contents of the paper. Ikuya Nonaka and Shin Yamada should not be listed as authors of the Article. The author list should read:

“Hiroko P. Indo, Hideyuki J. Majima, Masahiro Terada, Shigeaki Suenaga, Kazuo Tomita, Akira Higashibata, Noriaki Ishioka, Takuro Kanekura, Clare L. Hawkins, Michael J. Davies, Daret K. St Clair & Chiaki Mukai”

The Author Contribution statement should read:

“H.P.I., S.S., K.T. and T.K. contributed to the whole analyses and discussion of the results. M.T., C.L.H., M.D., D.K.S. and H.J.M. contributed to the experimental planning and discussion of the results. A.H., N.I., C.M., contributed to all of these aspects in this study, including drafting the manuscript and executing the final revisions. All authors approved the final manuscript prior to publication.”

The Acknowledgements should read:

“This study was supported in part by the JAXA-ISS Space Medicine Program Grant from the Japan Aerospace Exploration Agency. The authors gratefully thank the astronaut who took part in this study, Dr. Satoshi Furukawa and Dr. Shin Yamada of JAXA for their encouragement to complete this study, Dr. Ikuya Nonaka, National Center Hospital for Mental Nervous and Muscular Disorders, who contributed to start up this project, Professor John Tremarco of Kagoshima University for his help and guidance during the preparation of this manuscript.”

