# Supplementary material for: Changes in mitochondrial homeostasis and redox status in astronauts following long stays in space
Source: Sci Rep. 2016 Dec 16;6:39015. doi: 10.1038/srep39015 (PMC5159838; doi:10.1038/srep39015)
Supplement: Supplementary Figures [file srep39015-s1.pdf]

SREP-16-07925B **Supplementary Figures**

Title: **Changes in mitochondrial homeostasis and redox status in astronauts following long stays in space**

Authors: Hiroko P. Indo, Hideyuki J. Majima, Masahiro Terada, Shigeaki Suenaga, Kazuo Tomita, Shin Yamada, Akira Higashibata, Noriaki Ishioka, Takuro Kanekura, Ikuya Nonaka, Clare L. Hawkins, Michael J. Davies, Daret K. St Clair and Chiaki Mukai

Supplementary Fig. S1. The mtDNA/nDNA (1a), mtRNA/nRNA (1b) and mtRNA/mtDNA (1c) ratio changes at Preflight #1, #2, Inflight #1, #2 and Postflight #1, #2 for each astronaut. The hair samples taken from 10 astronauts were used for analysis. a: Astronaut 1. b: Astronaut 2. c: Astronaut 3. d: Astronaut 4. e: Astronaut 5. f: Astronaut 6. g: Astronaut 7. h: Astronaut 8. i: Astronaut 9. j: Astronaut 10. Upper left: Ratio of mtDNA/nDNA. *: $p \leq 0.05$ by ANOVA with Scheffe's F test, **: $p \leq 0.01$ by ANOVA with Scheffe's F test. Bar: SD.

Supplementary Fig. S2. Number of Increased and Decreased mtDNA/nDNA, mtRNA/nRNA and mtRNA/mtDNA ratio changes in 10 astronauts, at Preflight, Inflight and Postflight. The mtDNA/nDNA, mtRNA/nRNA and mtRNA/mtDNA ratios in each astronaut shown in Supplementary Fig. 1 and 2 are summarized. The numbers of significantly increased or decreased over all counted number of astronauts are shown. The calculated percentage(s) are also shown. For example, Preflight vs Inflight means increased or decreased ratio(s) when conditions of astronauts are changed to Inflight condition from

Preflight condition. *: $p \leq 0.05$ by ANOVA with Scheffe's F test, **: $p \leq 0.01$ by ANOVA with Scheffe's F test.

Supplementary Fig. S3. The MnSOD changes at Preflight #1, #2, Inflight #1, #2 and Postflight #1, #2 for each astronaut. The hair samples taken from 10 astronauts were used for analysis. a: Astronaut 1. b: Astronaut 2. c: Astronaut 3. d: Astronaut 4. e: Astronaut 5. f: Astronaut 6. g: Astronaut 7. h: Astronaut 8. i: Astronaut 9. j: Astronaut 10. *: $p \leq 0.05$ by ANOVA with Scheffe's F test, **: $p \leq 0.01$ by ANOVA with Scheffe's F test. Bar: SD.

Supplementary Fig. S4. The CuZnSOD expression changes at Preflight #1, #2, Inflight #1, #2 and Postflight #1, #2 for each astronaut. The hair samples taken from 10 astronauts were used for analysis. a: Astronaut 1. b: Astronaut 2. c: Astronaut 3. d: Astronaut 4. e: Astronaut 5. f: Astronaut 6. g: Astronaut 7. h: Astronaut 8. i: Astronaut 9. j: Astronaut 10. *: $p \leq 0.05$ by ANOVA with Scheffe's F test, **: $p \leq 0.01$ by ANOVA with Scheffe's F test. Bar: SD.

Supplementary Fig. S5. The Nrf2 expression changes at Preflight #1, #2, Inflight #1, #2 and Postflight #1, #2 for each astronaut. The hair samples taken from 10 astronauts were used for analysis. a: Astronaut 1. b: Astronaut 2. c: Astronaut 3. d: Astronaut 4. e: Astronaut 5. f: Astronaut 6. g: Astronaut 7. h: Astronaut 8. i: Astronaut 9. j: Astronaut 10. *: $p \leq 0.05$ by ANOVA with Scheffe's F test, **: $p \leq 0.01$ by ANOVA with Scheffe's F test. Bar: SD.

Supplementary Fig. S6. The Keap1 expression changes at Preflight #1, #2, Inflight #1, #2 and Postflight #1, #2 for each astronaut. The hair samples taken from 10 astronauts were used for analysis. a: Astronaut 1. b: Astronaut 2. c: Astronaut 3. d: Astronaut 4. e: Astronaut 5. f: Astronaut 6. g: Astronaut 7. h: Astronaut 8. i: Astronaut 9. j: Astronaut 10. *: $p \leq 0.05$ by ANOVA with Scheffe's F test, **: $p \leq 0.01$ by ANOVA with Scheffe's F test. Bar: SD.

Supplementary Fig. S7. The GPx4 expression changes at Preflight #1, #2, Inflight #1, #2 and Postflight #1, #2 for each astronaut. The hair samples taken from 10 astronauts were used for analysis. a: Astronaut 1. b: Astronaut 2. c: Astronaut 3. d: Astronaut 4. e: Astronaut 5. f: Astronaut 6. g: Astronaut 7. h: Astronaut 8. i: Astronaut 9. j: Astronaut 10. *: $p \leq 0.05$ by ANOVA with Scheffe's F test, **: $p \leq 0.01$ by ANOVA with Scheffe's F test. Bar: SD.

Supplementary Fig. S8. The Catalase expression changes at Preflight #1, #2, Inflight #1, #2 and Postflight #1, #2 for each astronaut. The hair samples taken from 10 astronauts were used for analysis. a: Astronaut 1. b: Astronaut 2. c: Astronaut 3. d: Astronaut 4. e: Astronaut 5. f: Astronaut 6. g: Astronaut 7. h: Astronaut 8. i: Astronaut 9. j: Astronaut 10. *: $p \leq 0.05$ by ANOVA with Scheffe's F test, **: $p \leq 0.01$ by ANOVA with Scheffe's F test. Bar: SD.

Supplementary Fig. S9. Combined data for the mtDNA/nDNA (9a), mtRNA/nRNA (9b) and mtRNA/mtDNA (9c) ratios from ten astronauts in comparison with Preflight #1, #2, Inflight #1, #2 and Postflight #1, #2. Mean data (+ standard errors of the mean) of the mtDNA/nDNA (9a), mtRNA/nRNA (9b) and mtRNA/mtDNA (9c) ratios for all 10 astronauts are shown. The mtRNA/mtDNA ratios shown in (9c) were calculated from data of mtRNA/nRNA and mtRNA/mtDNA according to the equation as shown in Materials and method. *: $p \leq 0.05$ by ANOVA with Scheffe's F test, **: $p \leq 0.01$ by ANOVA with Scheffe's F test.

Supplementary Fig. S10. Combined data for MnSOD expression changes in comparison with Preflight #1, #2, Inflight #1, #2 and Postflight #1, #2. Mean data (+ standard errors of the mean) of MnSOD expression change for all 10 astronauts are shown. *: $p \leq 0.05$ by ANOVA with Scheffe's F test, **: $p \leq 0.01$ by ANOVA with Scheffe's F test.

Supplementary Fig. S11. Combined data for CuZnSOD expression changes in comparison with Preflight #1, #2, Inflight #1, #2 and Postflight #1, #2. Mean data (+ standard errors of the mean) of CuZnSOD expression change for all 10 astronauts are shown. *: $p \leq 0.05$ by ANOVA with Scheffe's F test, **: $p \leq 0.01$ by ANOVA with Scheffe's F test.

Supplementary Fig. S12. Combined data for Nrf2 expression changes in comparison

with Preflight #1, #2, Inflight #1, #2 and Postflight #1, #2. Mean data (+ standard errors of the mean) of Nrf2 expression change for all 10 astronauts are shown. *: $p \leq 0.05$ by ANOVA with Scheffe's F test, **: $p \leq 0.01$ by ANOVA with Scheffe's F test.

Supplementary Fig. S13. Combined data for Keap1 expression changes in comparison with Preflight #1, #2, Inflight #1, #2 and Postflight #1, #2. Mean data (+ standard errors of the mean) of Keap1 expression change for all 10 astronauts are shown. *: $p \leq 0.05$ by ANOVA with Scheffe's F test, **: $p \leq 0.01$ by ANOVA with Scheffe's F test.

Supplementary Fig. S14. Combined data for GPx4 expression changes in comparison with Preflight #1, #2, Inflight #1, #2 and Postflight #1, #2. Mean data (+ standard errors of the mean) of GPx4 expression change for all 10 astronauts are shown. *: $p \leq 0.05$ by ANOVA with Scheffe's F test, **: $p \leq 0.01$ by ANOVA with Scheffe's F test.

Supplementary Fig. S15. Combined data for Catalase expression changes in comparison with Preflight #1, #2, Inflight #1, #2 and Postflight #1, #2. Mean data (+ standard errors of the mean) of Catalase expression change for all 10 astronauts are shown. *: $p \leq 0.05$ by ANOVA with Scheffe's F test, **: $p \leq 0.01$ by ANOVA with Scheffe's F test.

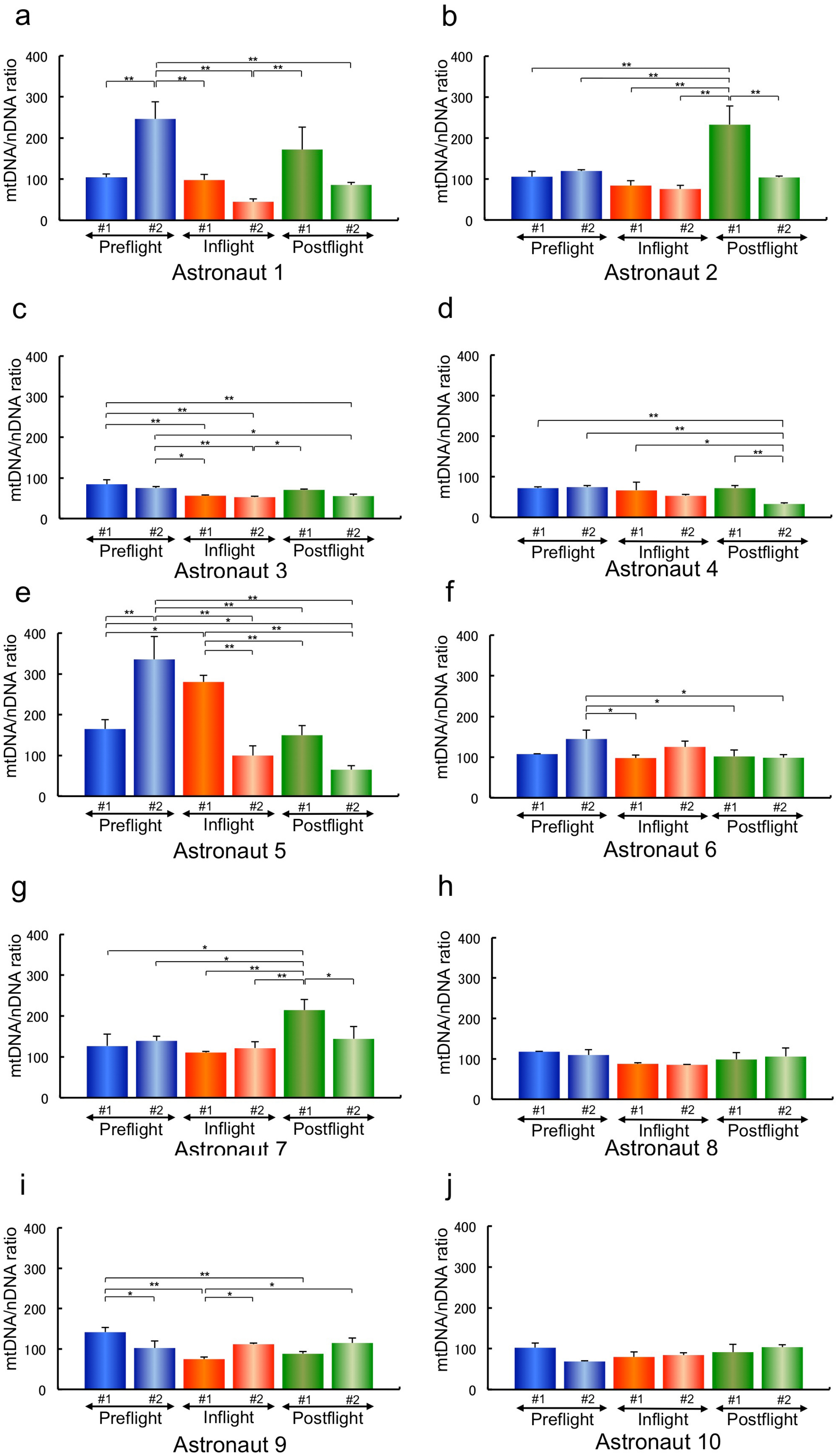

a
b
c
d
e
f
g
h
i
j
mtDNA/nDNA ratio
0
100
200
300
400
#1
#2
Preflight
Inflight
Postflight
Astronaut 1
Astronaut 2
Astronaut 3
Astronaut 4
Astronaut 5
Astronaut 6
Astronaut 7
Astronaut 8
Astronaut 9
Astronaut 10

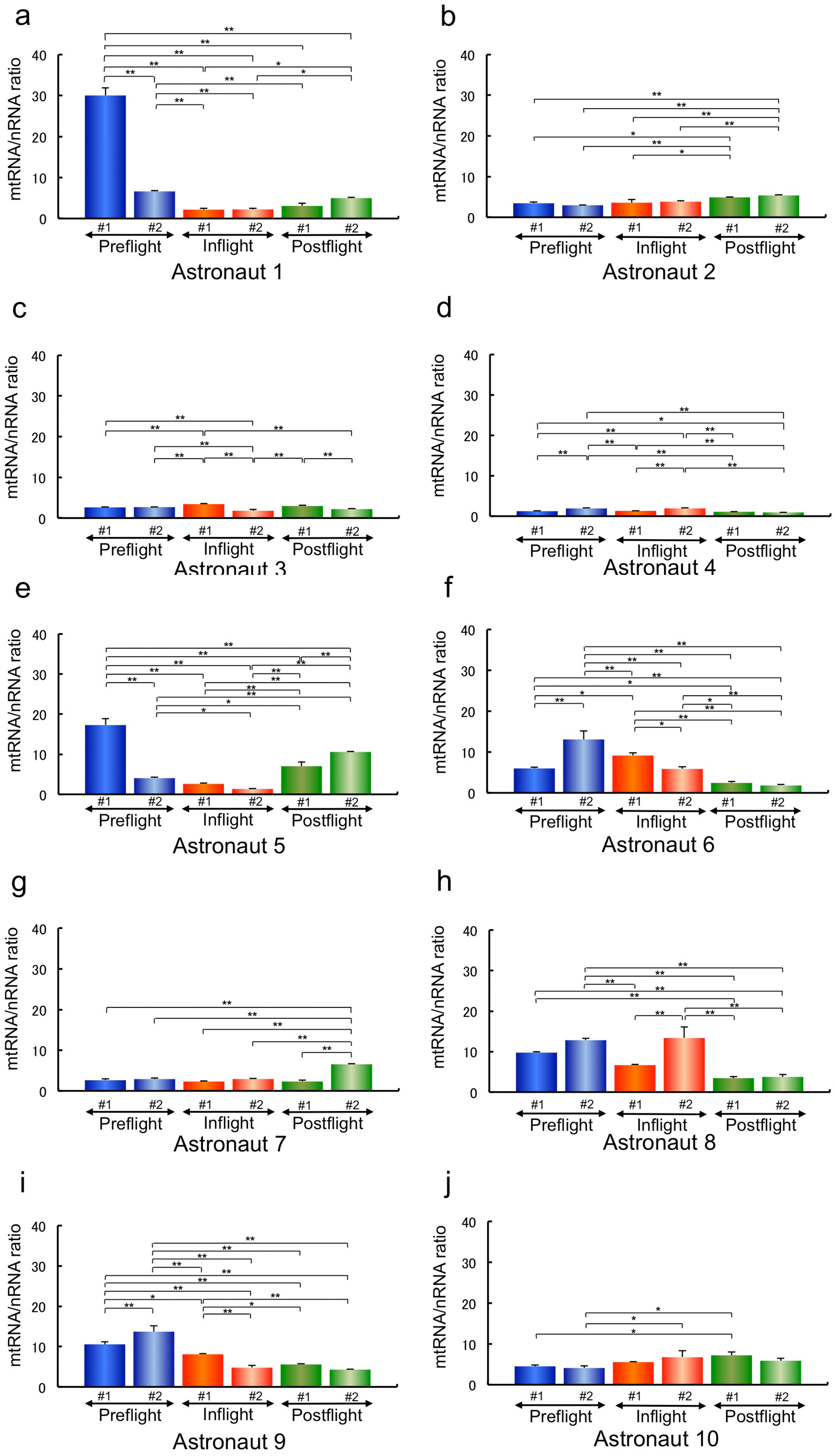
a
b
c
d
e
f
g
h
i
j
mtRNA/nRNA ratio
0
10
20
30
40
#1
#2
Preflight
Inflight
Postflight
Astronaut 1
Astronaut 2
Astronaut 3
Astronaut 4
Astronaut 5
Astronaut 6
Astronaut 7
Astronaut 8
Astronaut 9
Astronaut 10

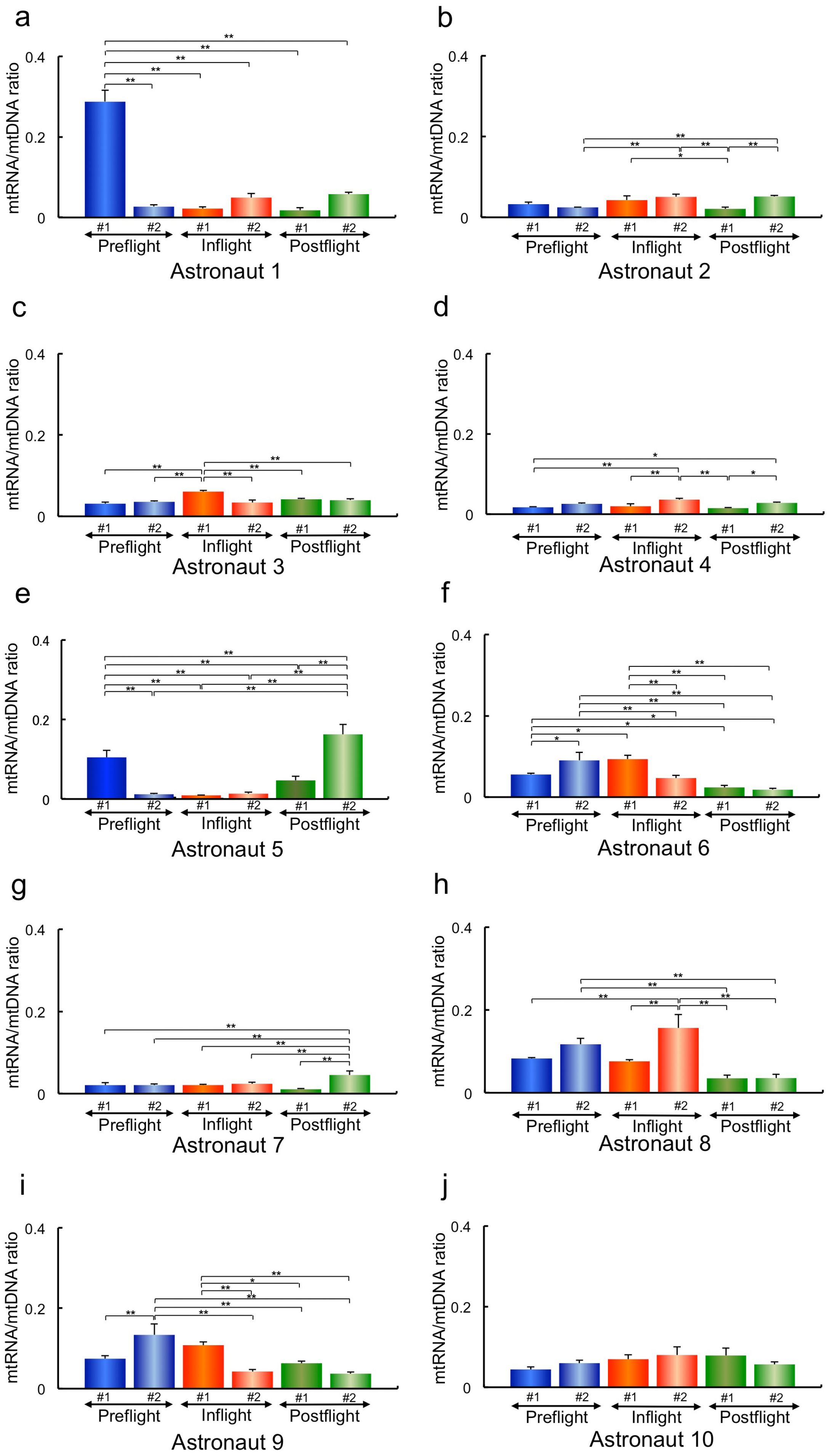

a
b
c
d
e
f
g
h
i
j
mtRNA/mtDNA ratio
0.4
0.2
0
#1
#2
Preflight
Inflight
Postflight
Astronaut 1
Astronaut 2
Astronaut 3
Astronaut 4
Astronaut 5
Astronaut 6
Astronaut 7
Astronaut 8
Astronaut 9
Astronaut 10

Each astronauts		mtDNA/nDNA ratios	mtRNA/nRNA ratios	mtRNA/mtDNA ratios
Preflight vs Inflight	Increased	1/10 (10% ∗:1, **:0)	4/10 (40% ∗:2, **:2)	5/10 (50% ∗:1, **:4)
	Decreased	5/10 (50% ∗:1, **:4)	7/10 (70% ∗:0, **:7)	4/10 (40% ∗:0, **:4)
Inflight vs Postflight	Increased	5/10 (50% ∗:2, **:3)	5/10 (50% ∗:1, **:4)	2/10 (20% ∗:0, **:2)
	Decreased	2/10 (20% ∗:1, **:1)	5/10 (50% ∗:0, **:5)	6/10 (60% ∗:0, **:6)
Preflight vs Postflight	Increased	2/10 (20% ∗:1, **:1)	4/10 (40% ∗:1, **:3)	4/10 (40% ∗:1, **:3)
	Decreased	6/10 (60% ∗:1, **:5)	6/10 (60% *:0, **:6)	5/10 (50% ∗:0, **:5)

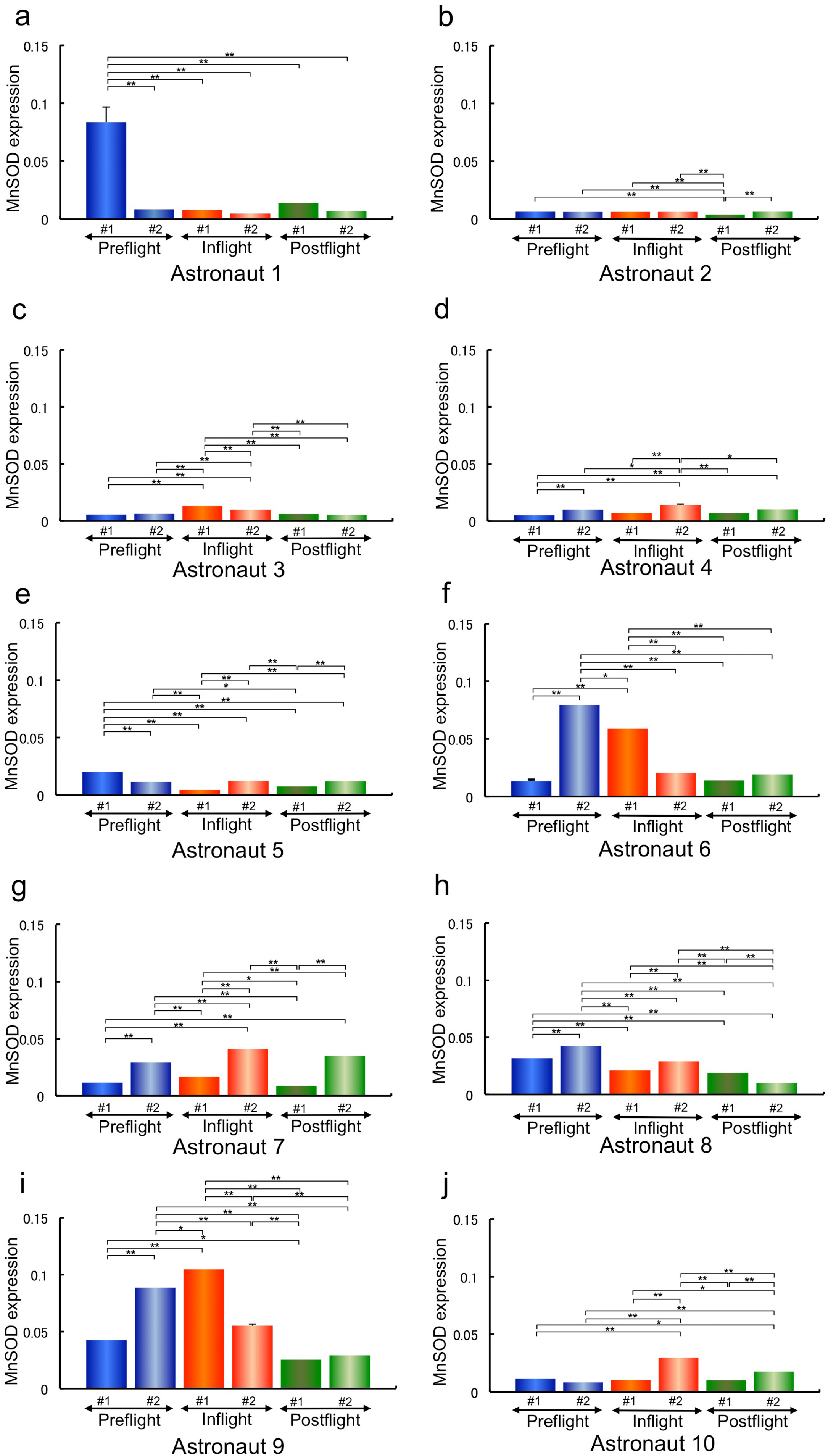

a
b
c
d
e
f
g
h
i
j
MnSOD expression
0
0.05
0.1
0.15
#1
#2
Preflight
Inflight
Postflight
Astronaut 1
Astronaut 2
Astronaut 3
Astronaut 4
Astronaut 5
Astronaut 6
Astronaut 7
Astronaut 8
Astronaut 9
Astronaut 10

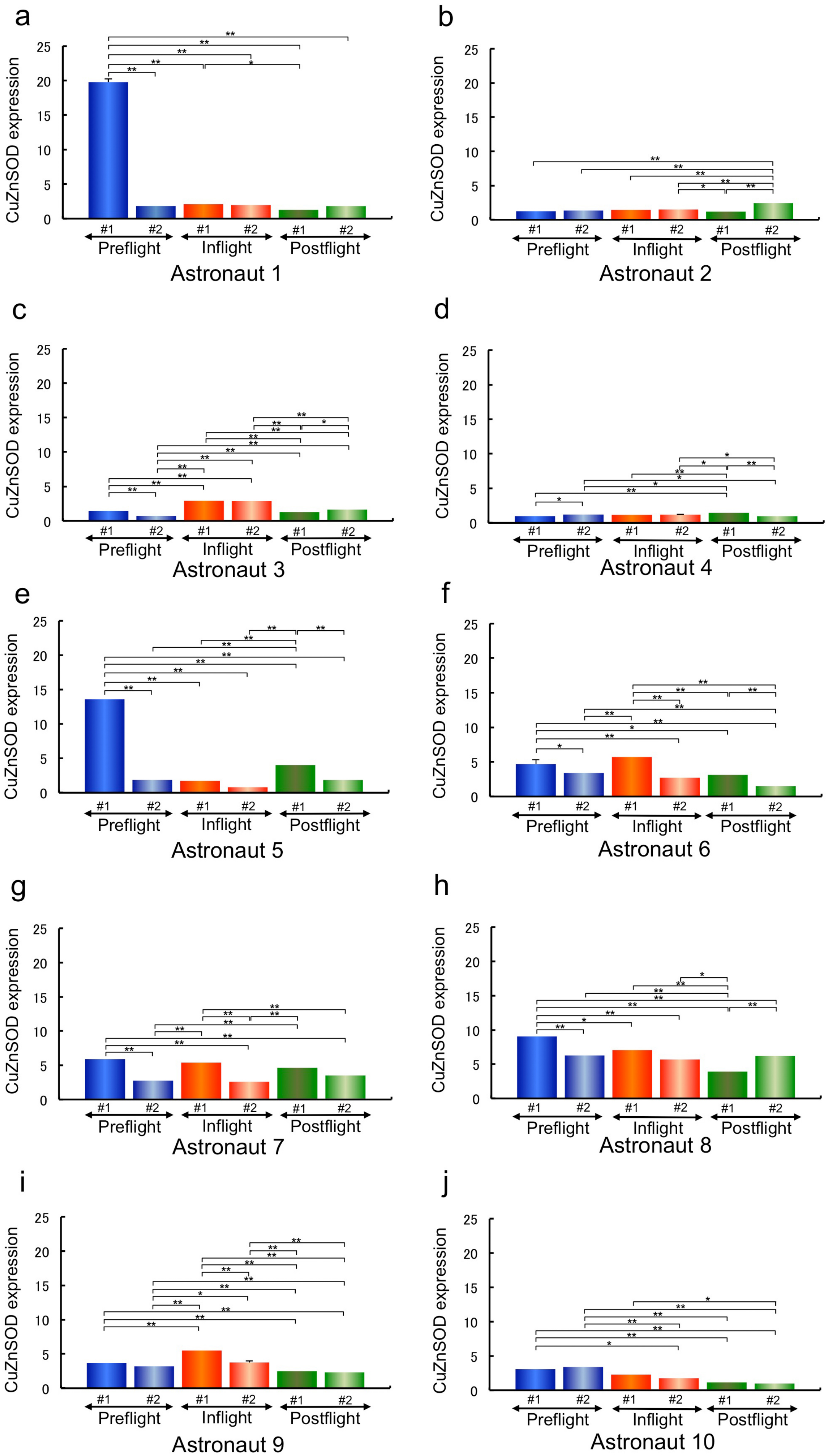
a
b
c
d
e
f
g
h
i
j
CuZnSOD expression
25
20
15
10
5
0
#1
#2
Preflight
Inflight
Postflight
Astronaut 1
Astronaut 2
Astronaut 3
Astronaut 4
Astronaut 5
Astronaut 6
Astronaut 7
Astronaut 8
Astronaut 9
Astronaut 10

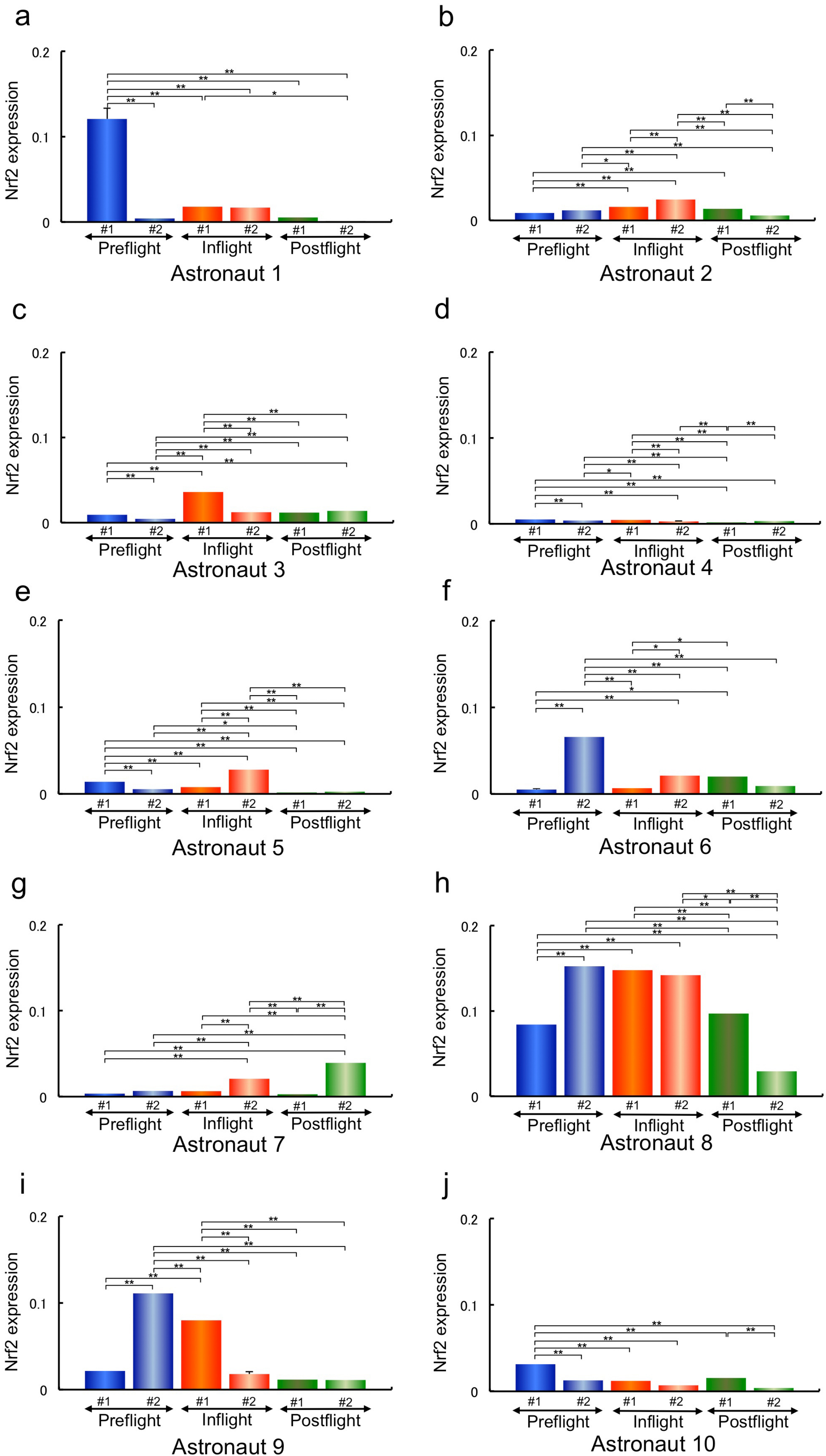
a
b
c
d
e
f
g
h
i
j
Nrf2 expression
0.2
0.1
0
#1
#2
Preflight
Inflight
Postflight
Astronaut 1
Astronaut 2
Astronaut 3
Astronaut 4
Astronaut 5
Astronaut 6
Astronaut 7
Astronaut 8
Astronaut 9
Astronaut 10

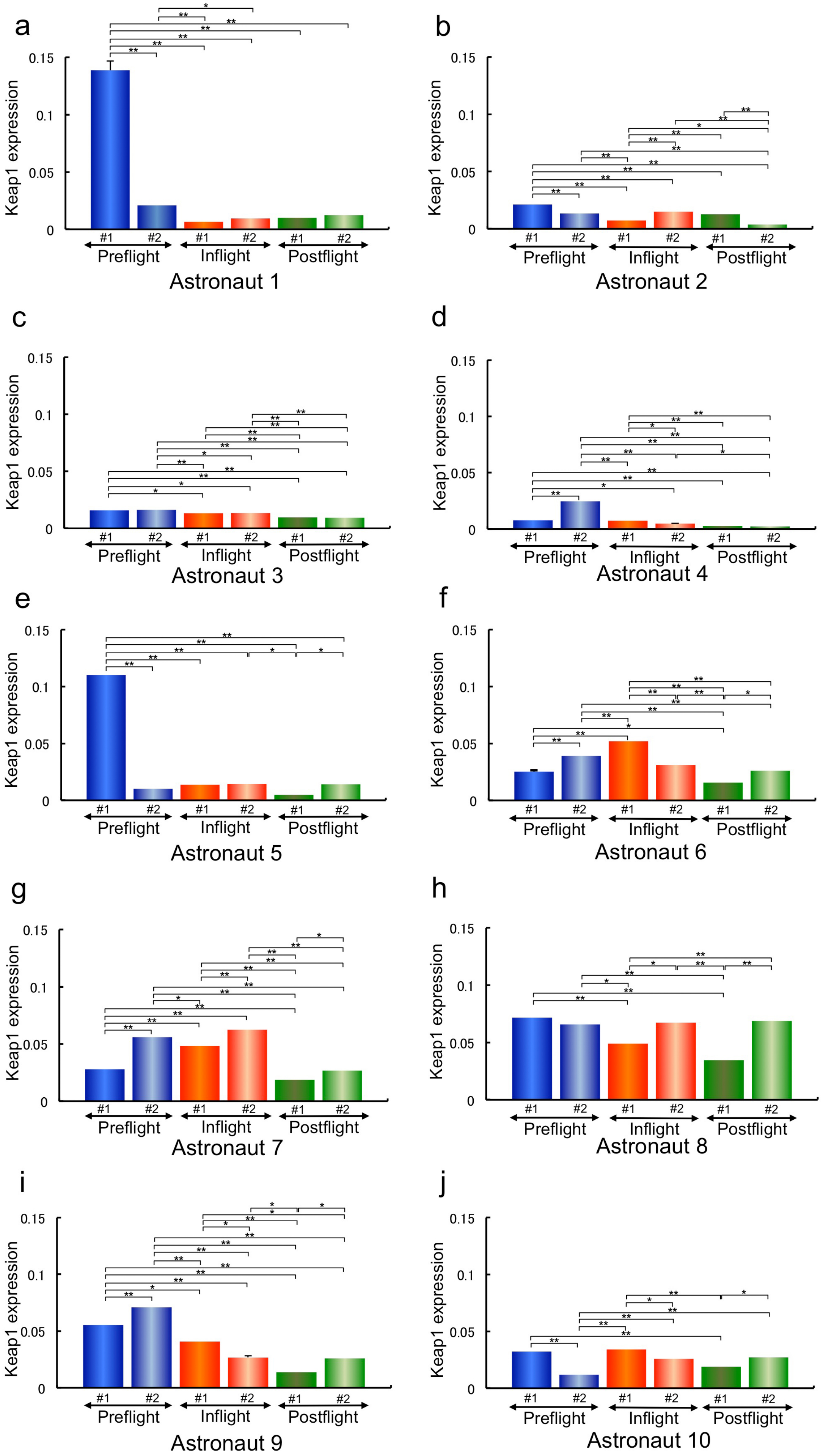

a
b
c
d
e
f
g
h
i
j
Keap1 expression
0
0.05
0.1
0.15
#1
#2
Preflight
Inflight
Postflight
*
**
Astronaut 1
Astronaut 2
Astronaut 3
Astronaut 4
Astronaut 5
Astronaut 6
Astronaut 7
Astronaut 8
Astronaut 9
Astronaut 10

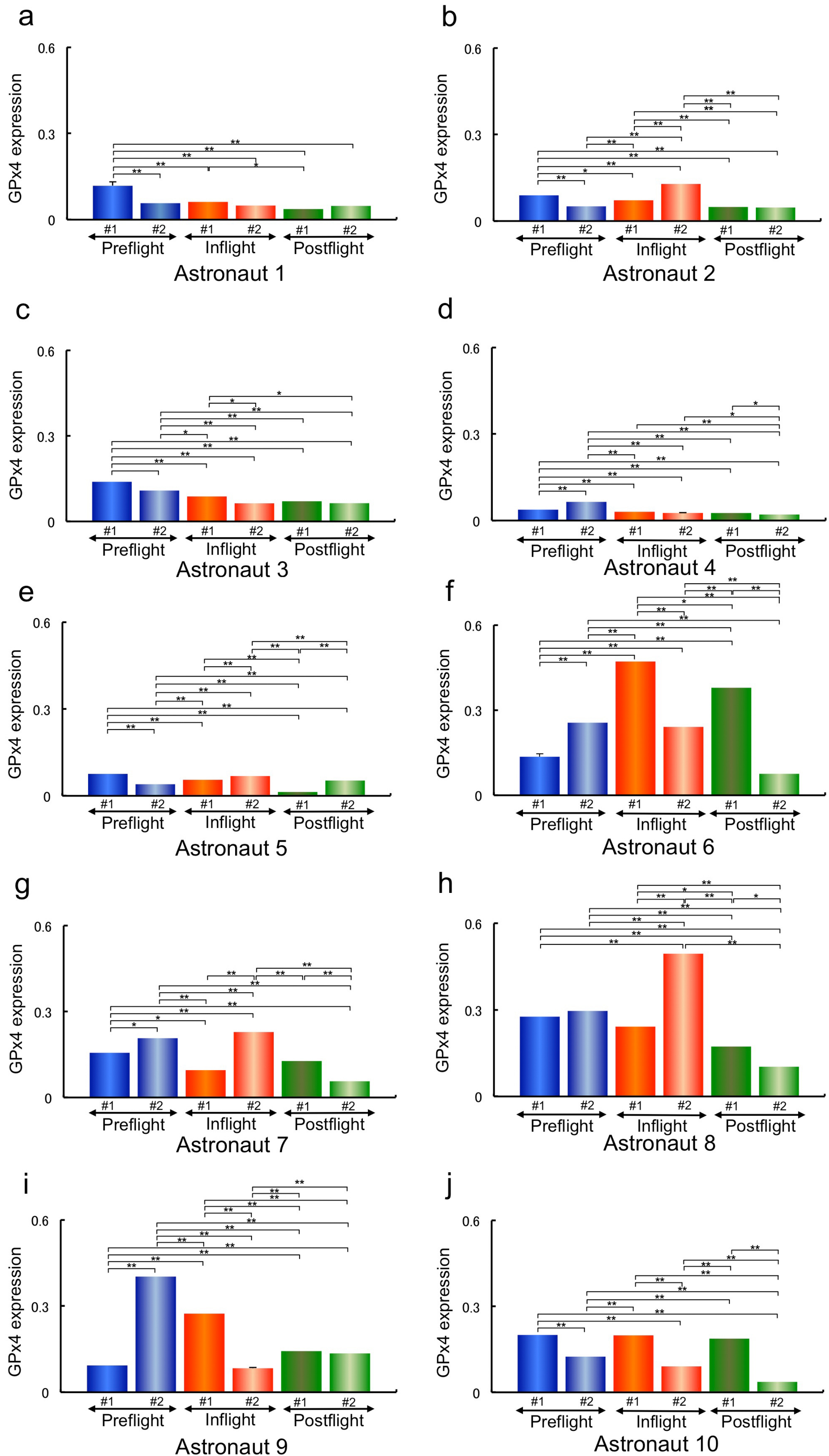

a
b
c
d
e
f
g
h
i
j
GPx4 expression
0
0.3
0.6
#1
#2
Preflight
Inflight
Postflight
Astronaut 1
Astronaut 2
Astronaut 3
Astronaut 4
Astronaut 5
Astronaut 6
Astronaut 7
Astronaut 8
Astronaut 9
Astronaut 10

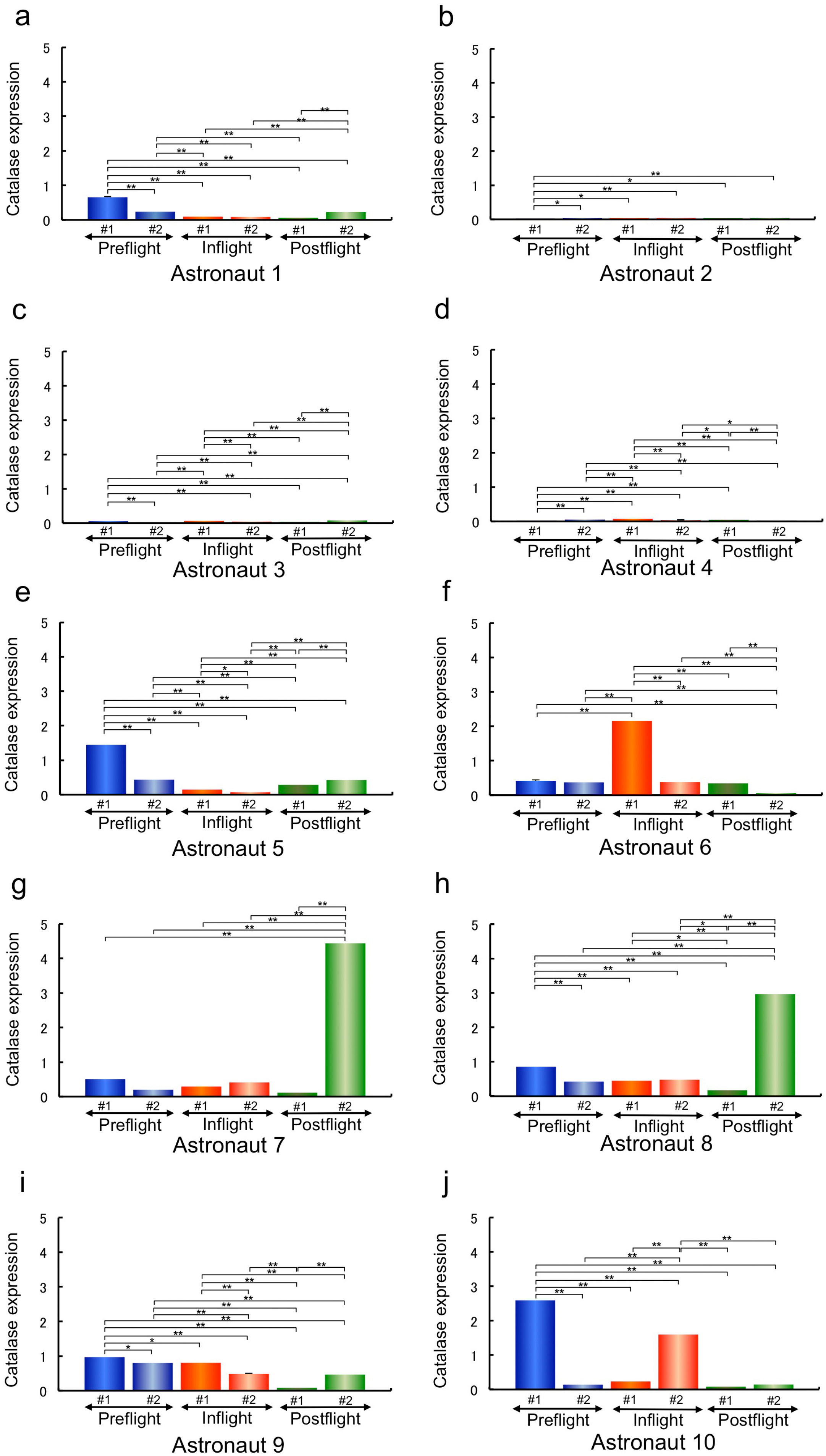

a
b
c
d
e
f
g
h
i
j
Catalase expression
0
1
2
3
4
5
#1
#2
Preflight
Inflight
Postflight
Astronaut 1
Astronaut 2
Astronaut 3
Astronaut 4
Astronaut 5
Astronaut 6
Astronaut 7
Astronaut 8
Astronaut 9
Astronaut 10

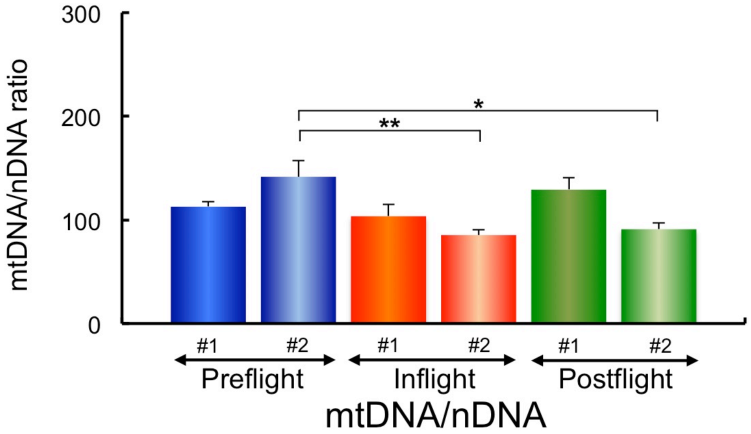

300
200
100
0
mtDNA/nDNA ratio
**
*
#1
#2
#1
#2
#1
#2
Preflight
Inflight
Postflight
mtDNA/nDNA

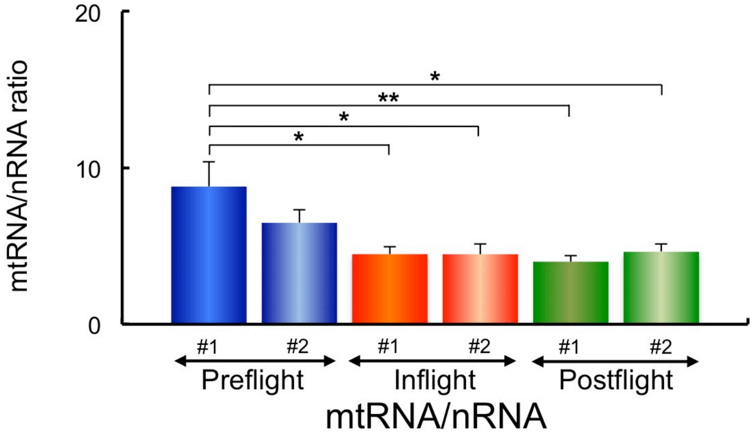

20
10
0
mtRNA/nRNA ratio
*
**
*
*
#1
#2
#1
#2
#1
#2
Preflight
Inflight
Postflight
mtRNA/nRNA

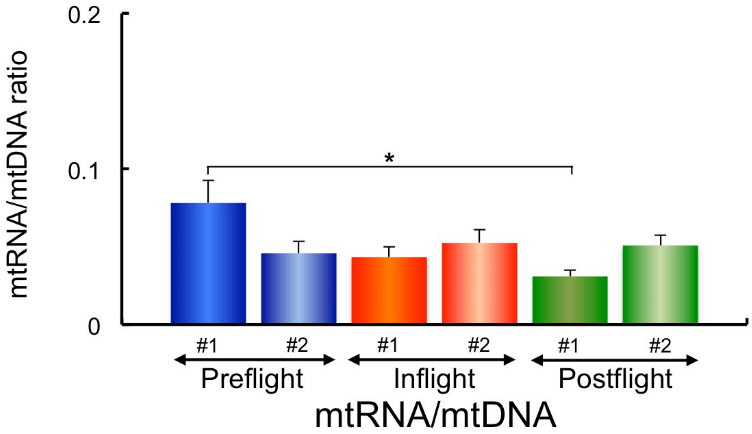
0.2
0.1
0
mtRNA/mtDNA ratio
*
#1
#2
#1
#2
#1
#2
Preflight
Inflight
Postflight
mtRNA/mtDNA

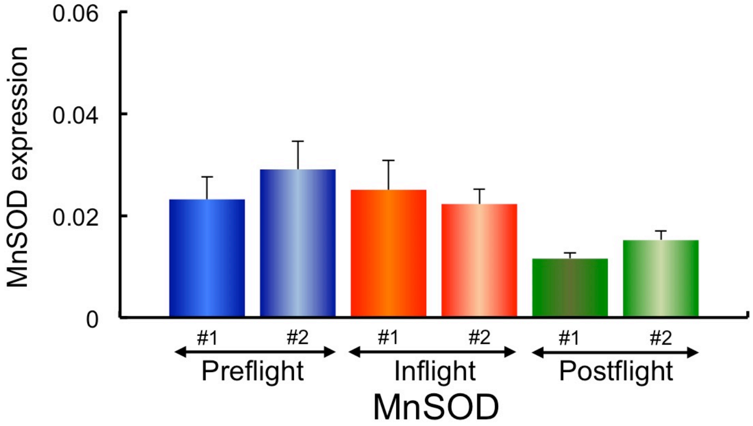
0.06
0.04
0.02
0
MnSOD expression
#1
#2
#1
#2
#1
#2
Preflight
Inflight
Postflight
MnSOD

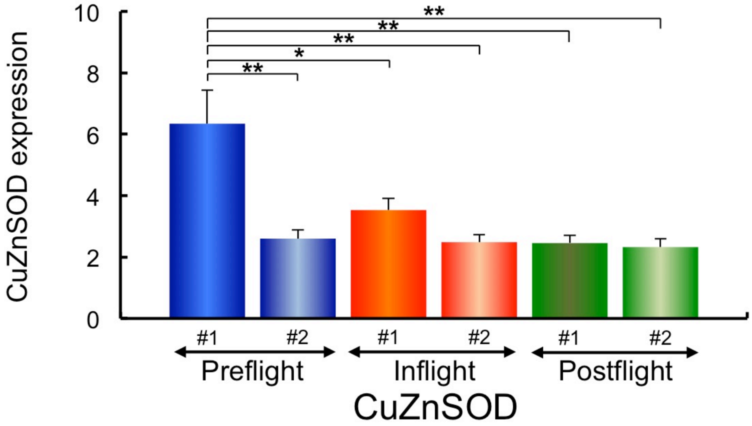
CuZnSOD expression
10
8
6
4
2
0
**
**
**
*
**
#1
#2
#1
#2
#1
#2
Preflight
Inflight
Postflight
CuZnSOD

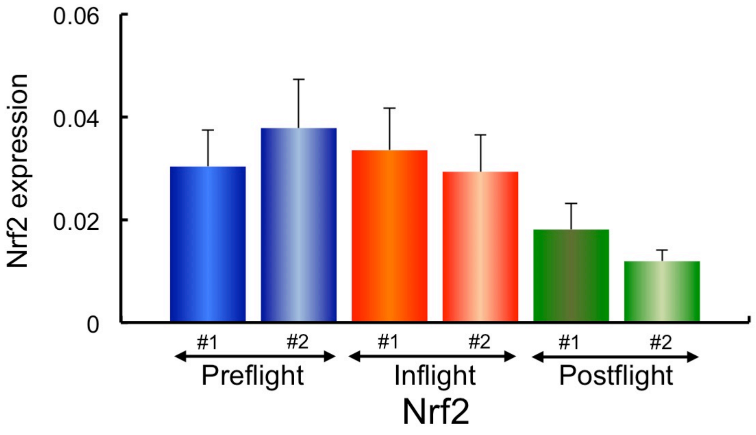

0.06
0.04
0.02
0
Nrf2 expression
#1
#2
#1
#2
#1
#2
Preflight
Inflight
Postflight
Nrf2

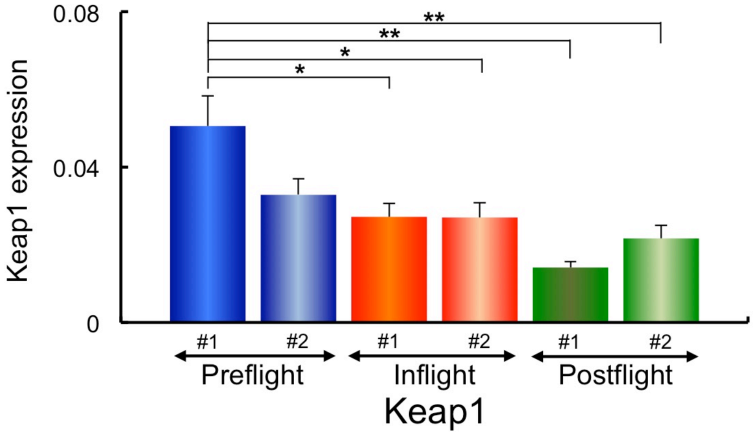

Keap1 expression
0.08
0.04
0
**
**
*
*
#1
#2
#1
#2
#1
#2
Preflight
Inflight
Postflight
Keap1

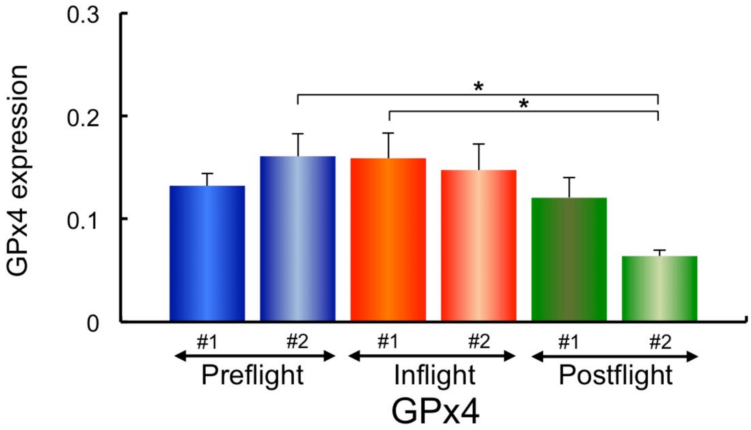
GPx4 expression
0.3
0.2
0.1
0
*
*
#1
#2
#1
#2
#1
#2
Preflight
Inflight
Postflight
GPx4

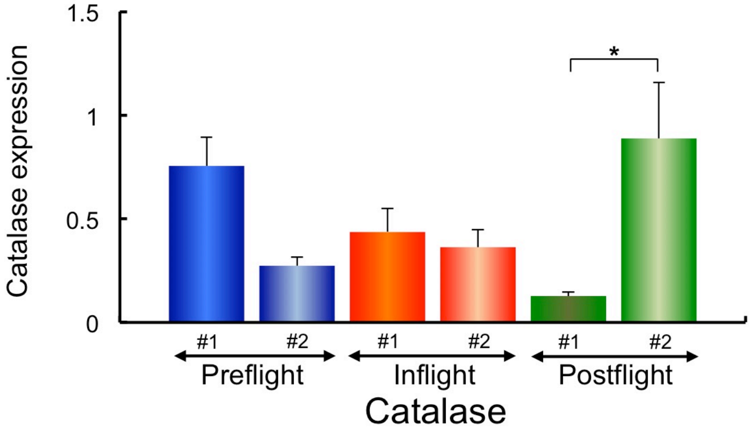
1.5
1
0.5
0
Catalase expression
*
#1
#2
#1
#2
#1
#2
Preflight
Inflight
Postflight
Catalase
